# Tuneable dielectric and optical characteristics of tailor-made inorganic electro-chromic materials

**DOI:** 10.1038/s41598-017-13941-9

**Published:** 2017-10-18

**Authors:** S. Bulja, R. Kopf, K. Nolan, R. Lundy, A. Tate, T. C. Hu, M. Norooziarab, R. Cahill, W. Templ

**Affiliations:** 1 Bell Labs, Nokia, Snugborough Road, Dublin, D15 Y6NT Ireland; 2 Bell Labs, 600 Mountain Ave., Murray Hill, NJ 07974 USA; 3 Queen’s University Belfast, The Institute of Electronics, Communications and Information Technology, Queen’s Road, BT3 9DT Belfast, UK; 4 Bell Labs Germany, Lorenzstrasse, 10, BW, 70435 Stuttgart, Germany

## Abstract

Electro-chromic materials (EC) are a new class of electronically reconfigurable thin films that have the ability to reversibly change optical properties by electric charge insertion/extraction. Since their discovery by Deb, they have been employed in applications related to display technology, such as smart windows and mirrors and active optical filters. In this sense, a variety of studies related to the tuneable optical characteristics of EC materials have recently been reported, however, their microwave tuneable dielectric characteristics have been left somewhat unexplored. In 2016 Bulja showed that dc bias voltage induced modulation of the optical characteristics of an inorganic Conductor/WO3/LiNbO3/NiO/Conductor EC cell isaccompanied by the modulation of its high frequency (1–20 GHz) dielectric characteristics. In general, according to the state of the art, cells of different material compositions are needed to produce devices of tailor made characteristics. Here, we report the discovery that the microwave dielectric and the optical characteristics of an EC cell can be engineered to suit a variety of applications without changing their material composition. The obtained results indicate the potential for producing novel, tuneable and tailor-engineered materials that can be used to create next generation agile microwave-optical devices.

## Introduction

The property of a change, evocation or bleaching of colour influenced by either an electron-transfer (redox) process or by a sufficient electrochemical potential, referred to as electrochromism, is exhibited by several organic and inorganic materials^1–14^. Depending on their physical state at room temperature, they can be clustered into three different types. Type I EC materials are soluble in the non-activated state and remain soluble after activation; type II EC materials are soluble in their non-activated state, but form a solid upon activation; and type III are EC materials which are solid in their non-activated and activated states.

**Figure 1:**
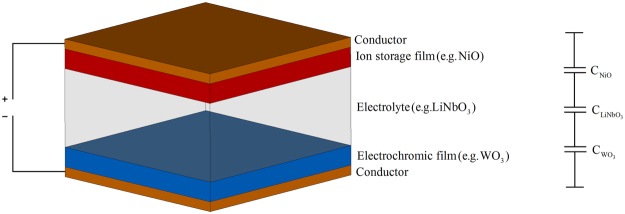
Basic structure of inorganic EC cell: (**a**) perspective view. (**b**) Capacitance model.

A basic structure of an EC cell is shown in Fig. 1(a). Here, glass (not shown for conciseness) is normally used as the substrate on which the individual layers of the EC material are deposited, as it provides structural stability and is permeable to light; however, other structurally stable substrates can also be used. The optically transparent conductors usually come in the form of Indium Tin Oxide (ITO), Zinc Oxide (ZnO) or a form of conductive polymers, and need to be good electrical conductors. The EC film for inorganic based EC materials at the cathode end is usually tungsten oxide (WO_3_), however, a variety of other transition metal oxides can be employed (TiO_2_, MoO_3_, Ta_2_O_5_, Nb_2_O_5_)^12,15–18^ depending on the desired colour of the “ON” or actuated state. It is this layer that contributes to colour modulation based on ion and electron injection. The ion conductor or electrolyte layer serves as a tank of available ions to be injected into the EC film(s). The requirement imposed on this layer is that it needs to display different ion and electron conductivities, typically ${\sigma }_{I} > {10}^{-7}S/\text{cm}\,$ for ions and ${\sigma }_{e} < {10}^{-10}S/\text{cm}\,$ for electrons^5,12^. The ion storage layer, if present, should display complementary electrochromic characteristics to the cathodic EC film. Typical transition metal oxides used for this layer are NiO, Cr_2_O_3_, MnO_2_, FeO_2_, CoO_2_, RhO_2_, and IrO_2_. The ability to tune the high frequency dielectric properties of an inorganic type III EC cell^19^ demonstrated not only the usability of EC materials outside of the traditional display-related technologies, but also it pointed at the possibility to develop a new family of devices capable of exploiting the pairing of the two phenomena. In this view^19^, EC cells with different material compositions (chromic layers and electrolytes), not only exhibit different tuneable optical characteristics, but, also different tuneable dielectric characteristics which are observed at microwave and millimetre wavelengths.

In this work we present first characterisation results on tailor-made tuneable microwave dielectric and optical characteristics of two inorganic, EC cells with complementary Conductor/WO_3_/LiNbO_3_/NiO/Conductor structure and same material composition. In particular, these two EC cells only differ in the thickness of the ion storage layer, whereas the composition and the thicknesses of both chromic layers are the same. Our results indicate that upon activation, even though both cells turn opaque, their exact dielectric and optical characteristics differ from each other. These encouraging initial results suggest the possibility of precise tailoring of the dielectric and optical responses of EC cells to suit a variety of microwave/millimetre-wave applications. Finally, we discuss the paper in context of its findings.

## EC Cell Measurement Device

**Figure 2:**
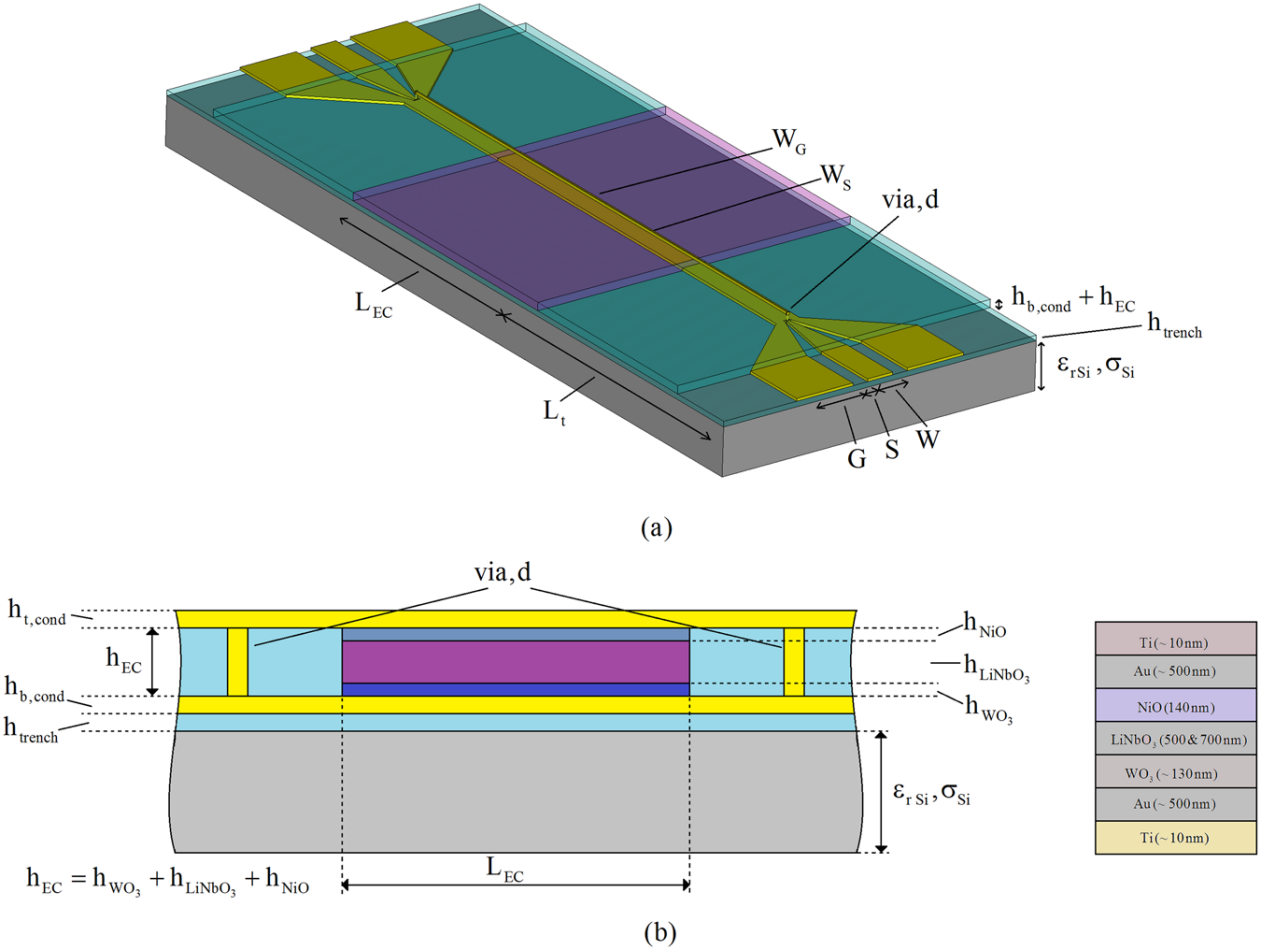
Schematic of EC measurement device. (**a**) Perspective view. (**b**) Side view and material layer stack.

Figure 2 ^20^ shows a schematic of the device used to measure both dielectric and optical characteristics of the EC cells. It also shows that the EC cell under test is enclosed by electrodes on the top and bottom surfaces, and at each end is terminated in a broadband impedance transformer. Here, the electrode plays the role of the anode of the thus formed EC cell. The two broadband impedance transformers also act as transitions from the anode to the coplanar waveguide (CPW), needed for on-wafer measurements. This improved version of the measurement device, considers the experiences related to the conductor thickness and electrodes shape from our preceding work^19^. In particular, the thickness of the top electrode is now reduced from 2.5 µm to 0.5 µm, the width of the bottom electrode is shaped to suppress the spurious resonances observed in the direction perpendicular to wave propagation and the impedance-matching of the transition section has been improved in order to minimise signal reflections at the boundary between the coplanar waveguide and the microstrip line. Two EC test devices are fabricated, differing only in the height of the LiNbO_3_ electrolyte layer, h_LiNbO3_.

The devices were fabricated using standard processing techniques that are fully compatible with semiconductor device manufacturing processes. The bottom substrate of the device structure shown in Fig. 2 (not to scale), with a height of 600 µm is an n-doped Si wafer with a surface resistivity of 10 Ω.cm and dielectric constant of ε_rSi_ = 11.9. On top of this layer, a thin dielectric layer of SiO_2_ with a height of h_trench_ = 300 nm is deposited so as to separate the conductive Si layer from the deposited gold ground plane. The SiO_2_ layer was deposited using Plasma-Enhanced Chemical-Vapor-Deposition in a Plasma-Therm Shuttle-lock system. It was then lithographically patterned using positive resist on a Suss contact aligner and was subsequently etched using Inductively- Coupled Reactive-Ion Plasma Etching in an SLR770 PlasmaTherm etcher to expose the contact pads and open the vias. The thickness of the ground plane is h_b,cond_ = 520 nm and it contains the patterned CPW. The dimensions of the CPW are G = 180 µm, S = 44 µm and W = 80 µm, and the length of the broadband transition is L_t_ = 2030 µm. The width of the top electrode and, hence, the anode of the EC cell is W_EC_ = 4 µm and its length is L_EC_ = 2 mm. The top electrode is gold, deposited partially on the SiO_2_ substrate and partially on the composite EC material, consisting of WO_3_, NiO and LiNbO_3_. The WO_3_, NiO and LiNbO_3_ layers were patterned using positive resist on a Suss contact aligner. The resist was then image-reversed to obtain a re-entrant profile for good lift-off. These layers were deposited using e-beam evaporation in an Airco-Temescal evaporator. The evaporator is equipped with an external O_2_ source, which is used during deposition of the WO_3_, NiO and LiNbO_3_ layers to maintain proper stoichiometry. These oxide layers are most likely amorphous, however, X-ray crystallography has not been performed on them to determine this. A 10 nm Ti layer (not shown in the figure) was used as an adhesion promoter for the WO_3_ layer to the golden ground plane.

**Figure 3:**
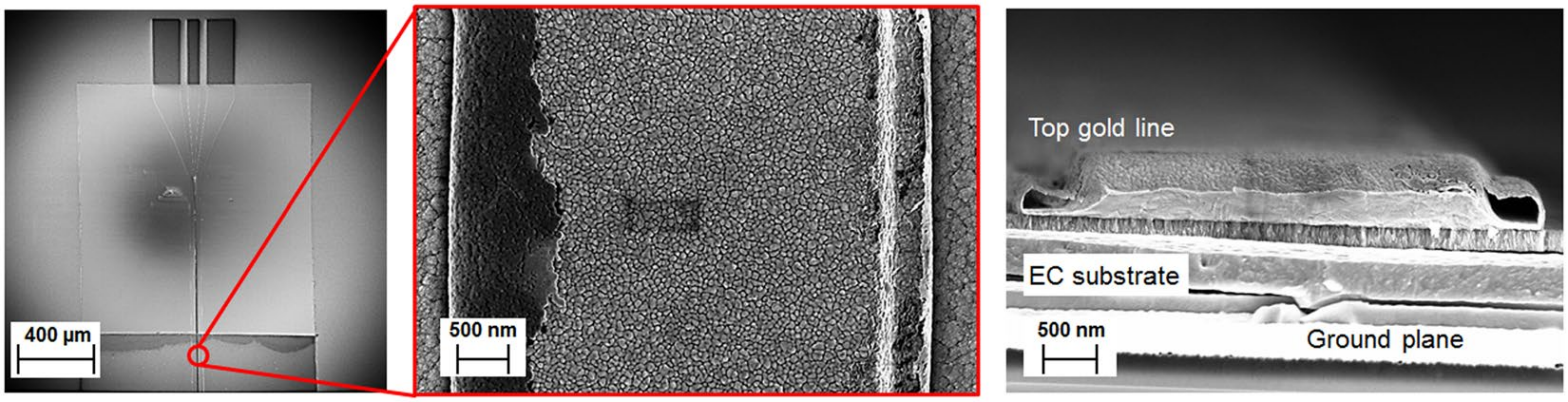
Field Emission Scanning Electron Microscopy (FESEM) of EC based device. (**a**) Top view of the fabricated EC measurement device. The input CPW pads are visible at the top. In the centre and at the bottom the SiO_2_ and EC layers are visible. (**b**) Top view of the gold electrode deposited on top of the composite EC substrate. (**c**) Cleaved image of the EC cell, showing the top gold line, EC substrate and the ground plane.

The thicknesses of the constituent chromic layers of the EC cell, namely WO_3_ and NiO layers, were chosen in accordance with the relevant previously reported work^11,12^ shown to exhibit the electro-chromic effect. In this study, the respective thicknesses of these layers are h_WO3_ = 130 nm and h_NiO_ = 140 nm. All of the individual layers were characterized using Stylus profilometry on a KLA Tencore P-11 profilometer to determine the thickness. In addition, ellipsometry and interferometry were used to determine the refractive indices and the thicknesses of the individual WO_3_, NiO and LiNbO_3_ layers prior to actual device fabrication. The obtained refractive indices were in agreement with published values. The heights of the LiNbO_3_ layers for the two different EC cells are h_LiNbO3,1_ = 500 nm (cell 1) and h_LiNbO3,2_ = 700 nm (cell 2), which render the overall heights of the two EC cells h_EC,1_ = h_WO3_ + h_LiNbO3,1_ + h_NiO_ = 770 nm (cell 1) and h_EC,2_ = h_WO3_ + h_LiNbO3,2_ + h_NiO_ = 970 nm (cell 2). Figure 3 shows images of the finished devices made by Field Emission Scanning Electron Microscopy (FESEM, Carl Zeiss Ultra) using a secondary electron detector at an accelerating voltage of 4 kV.

## Results

On-wafer measurements of the high frequency characteristics (scattering parameters) of the two structures containing the EC cells were performed using a Rohde-Schwarz Vector Network Analyser (VNA), over the frequency range of 1 GHz–20 GHz at an input power level of 5 dBm. A dc voltage bias needed for the modulation of the optical and dielectric characteristics is applied through one of the bias tees of the VNA. Typical dc bias voltages needed for the actuation of the inorganic EC cell are about 3–10 V, depending on its thickness. Here, we have experimentally verified that cell 2 (970 nm thick) fully switches on at 9 V, while cell 1 fully switches on at a dc bias voltage of about 7.1 V. The determination of the exact dc bias switching voltage for both cells was obtained in a systematic manner. Initially, the applied dc bias voltage was increased in steps of 1 V and the cells were kept at this dc bias voltage for 6 minutes, while their responses were monitored. This procedure was continued up to the dc bias voltage point when no changes in the magnitude of the transmission coefficient were observed. At this point (7 V for cell 1 and 9 V for cell 2), the step of the dc bias was refined to 0.1 V in order to capture the exact dc bias voltage at which the EC cells fully switch on (saturates). For cell 1, this voltage was recorded to be V_dc,1_ = 7.1 V and for cell 2 V_dc,2_ = 9 V. In general, the applied dc bias voltage needed for proper operation of EC cells is dependent on many factors, but it is usually between 3 V and 10 V. The main parameters that influence the dc bias voltage are the thicknesses of the constituent layers, chemical composition of the layers and the material purity of the layers.

**Figure 4:**
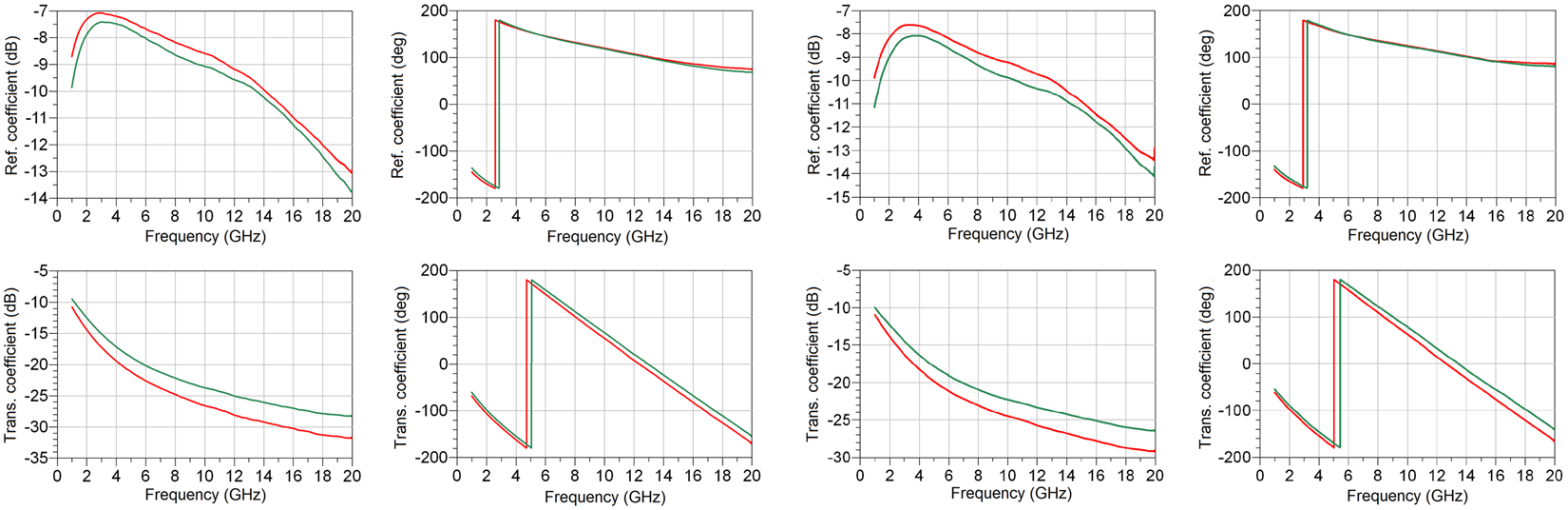
Measured scattering parameters of two structures containing EC cells. Green line corresponds to the unbiased state (Vdc = 0 V) and red line corresponds to the fully switched-on state (V_dc1,2_ = 7.1V, 9 V). **(a**) Cell 1 with h_LiNbO3,1_ = 500 nm, V_dc,1_ = 7.1 V and (**b**), Cell 2 with h_LiNbO3,2_ = 700 nm, V_dc,2_ = 9 V.

**Figure 5:**
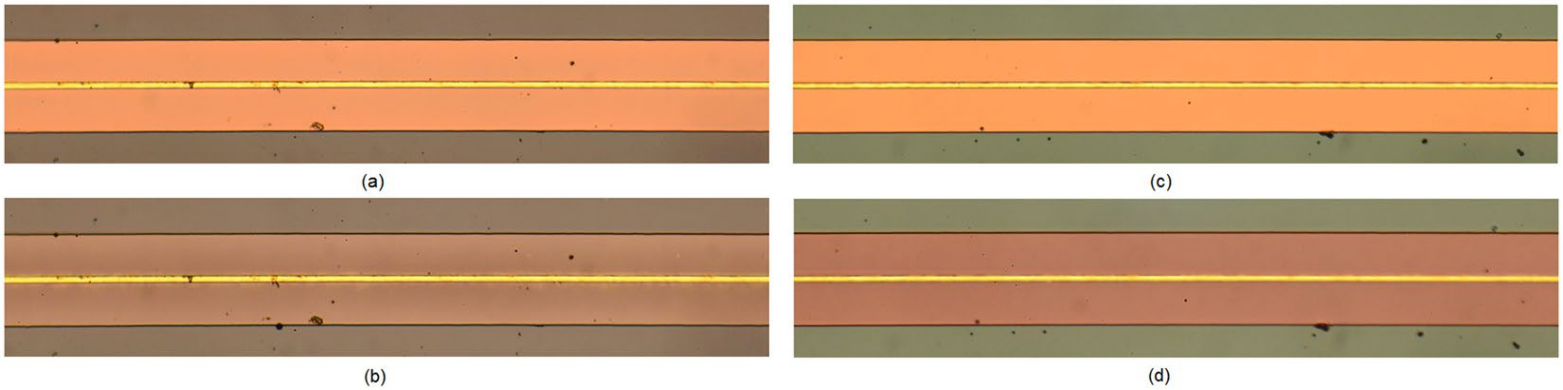
Photographs showing the measured colour change of the EC cells. (**a**) Cell 1 in the unbiased state (0 V). (**b**) Cell 1 in the biased state (7.1 V). (**c**) Cell 2 in the unbiased state (0 V). (**d**) Cell 2 in the biased state (9 V).

Figure 4 shows the measured scattering parameters obtained from the two structures containing the EC cells for the unbiased (green) and fully biased (red) states. The corresponding change in optical transmission colour of the two EC cells is shown in Fig. 5. The figure indicates that both cells exhibit an appreciable change in colour upon actuation by dc bias voltage, which is not limited to the region immediately below the top electrode; rather it spreads across the entire width of the EC cells with its boundaries defined by the width of the bottom ground plane. This is due to the fact that the chromic WO_3_ and NiO layers are deposited across the width of the entire ground plane and intensify their colour upon ion intercalation.

**Figure 6:**
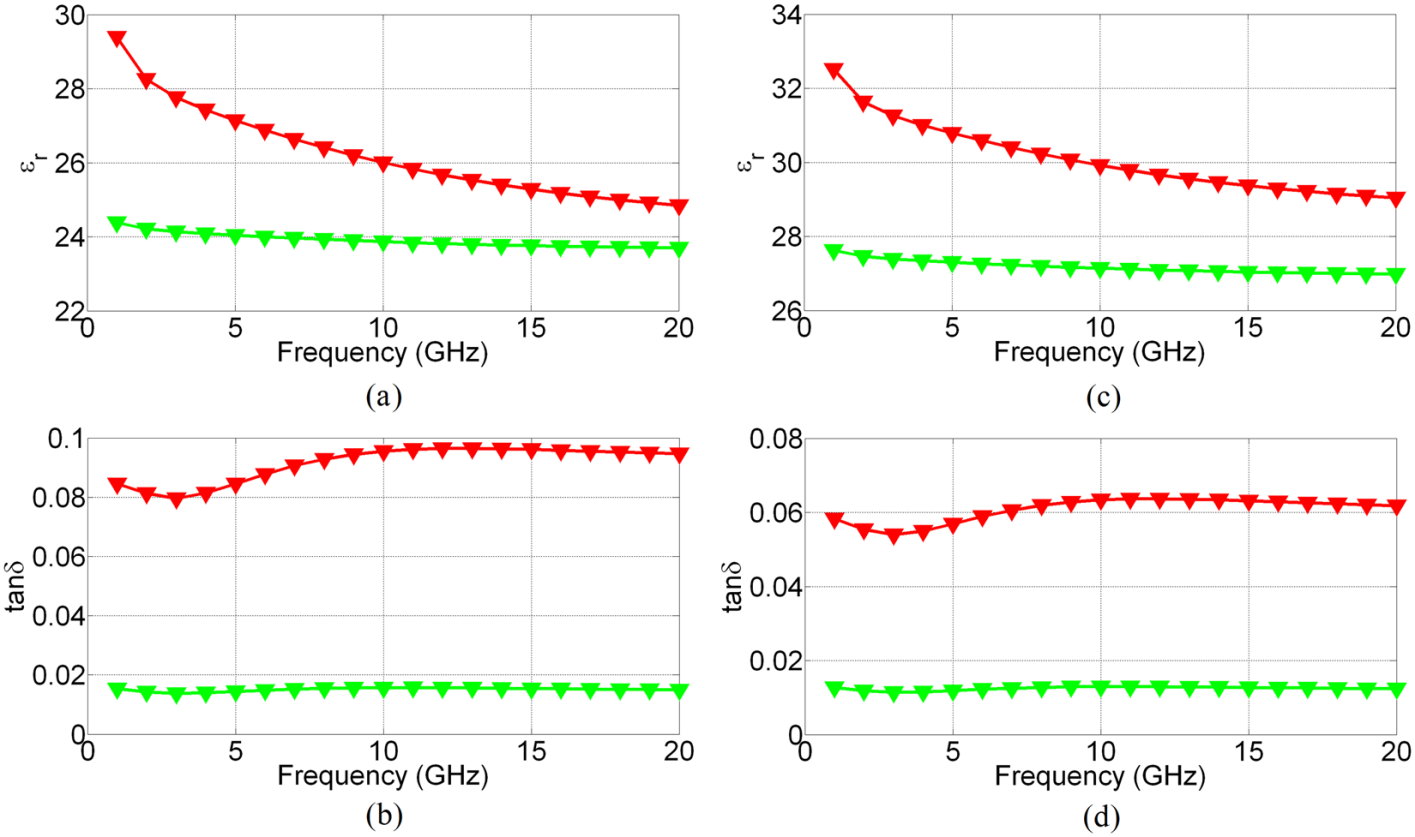
Extracted dielectric characteristics of the two EC cells: dielectric permittivity (**a**) and loss tangent (**b**) of cell 1, dielectric permittivity (**c**) and loss tangent (**d**), of cell 2. Green line corresponds to the unbiased state (Vdc = 0 V) and red line corresponds to the fully switched-on state (V_dc1,2_ = 7.1 V, 9 V).

In order to extract the unknown parameters (permittivity and loss tangent) of cell 1 and cell 2 from the measured scattering parameters of the EC cell structures shown in Fig. 4, the effect of the CPW-to-microstrip transitions shown in Figs 1a and 2a are extracted first. The de-embedding is performed using a TL^21^ procedure to yield the propagation constant of the EC cell

1 $${\gamma }_{EC}=\frac{1}{{L}_{EC}}cos{h}^{-1}(\frac{1+{T}_{{11}_{EC}}^{2}-{T}_{{21}_{EC}}^{2}}{2{T}_{{11}_{EC}}})$$

where *L*_*EC*_ depicted in Fig. 2(a) is the length of the top electrode exposed to the EC material, and ${T}_{{11}_{EC}}$, ${T}_{{21}_{EC}}\,$ are the elements of the transmission matrices of the EC cell obtained using said TL^21^ procedure. The propagation constant given by (1) contains information not only on the unknown dielectric properties of the EC material, but also includes the effect of the electrically thin bottom and top electrodes. Due to their low electrical thickness, the electrodes not only introduce additional losses but also allow EM wave propagation through their interior and subsequently into the air, which has a major impact on the extracted dielectric permittivity of the EC cells. This effect cannot be adequately considered analytically, however, by having an exact knowledge of the geometry of the EC cell, the unknown dielectric parameters can be numerically extracted. In conjunction with the exact physical dimensions of the top electrode and the thickness of the EC cells obtained by using FESEM and a Finite Integration Technique available in a 3D numerical simulator^22^ it is possible to accurately extract the exact dielectric parameters of the EC cells. With reference to Fig. 3(c), a particular challenge with numerical extraction lies with appropriately taking into account the edges of the top electrode. The lift-off process for this layer is not optimized resulting in curled conductive “flags” on the top electrode. This geometry results in the creation of an unwanted wave-guide effect, bounded by electrically thin conductors. The measured thickness of the conductor forming the waveguide on the left-hand side of Fig. 3(c) is approximately 40 nm, which at the frequencies of interest (1 GHz–20 GHz), results in increased losses and increased effective dielectric permittivity, given by (1). This was adequately addressed by slicing the EC cells at several positions to determine the average size and geometry of the top electrode. This information is then used in the numerical simulator to obtain the precise values of the dielectric characteristics of the unknown EC material. The extracted dielectric parameters, ${\varepsilon }_{r}$ and $\tan (\delta )$, of the EC cells are shown in Fig. 6. It can be appreciated from this figure that upon the application of a dc bias voltage, modulation of the dielectric parameters (dielectric permittivity and loss tangent) of both cells has taken place, however, the absolute values of the dielectric constants, loss tangents and their tunability are different for the two cells. In particular, dielectric tunability, calculated as ${\rm{\Delta }}\varepsilon =\frac{{\varepsilon }_{rON}-\,{\varepsilon }_{rOFF}}{{\varepsilon }_{rOFF}}\times 100 \%$, for cell 1 stands at 20.5 % and 4.8 % at frequencies of 1 GHz and 20 GHz respectively. Similarly, cell 2 has a dielectric tunability of 17.7 % and 7.6 % at frequencies of 1 GHz and 20 GHz respectively. The tunability of the loss tangent of both cells, calculated as ${\rm{\Delta }}\,\tan (\delta )=\frac{\tan \,{(\delta )}_{ON}-\,\tan \,{(\delta )}_{OFF}\,}{\tan \,{(\delta )}_{OFF}}\times 100 \%$, is much larger than that of the dielectric permittivity. For cell 1 it stands at 450 % and 530 % at frequencies of 1 and 20 GHz respectively, while the tunability of the loss tangent of cell 2 is 358 % and 400 % at the indicated frequency points. The absolute values of dielectric constant tunability compare favourably with a corresponding bulk material tuneable technology such as liquid crystals^23–26^ (LCs), which exhibit a typical tunability of the dielectric constant of up to 27 %^27^, with the values of the dielectric constants in the range of ${\varepsilon }_{{r}_{perp}}=2.47\ldots 2.82$ and ${\varepsilon }_{{r}_{par}}=3.18\ldots 3.38$. The only commercially available LC mixture, GT3 23001^27^, exhibits ${\varepsilon }_{{r}_{perp}}=2.47$ and ${\varepsilon }_{{r}_{par}}=3.25$ in the frequency range 140–165 GHz. The absolute values of the loss tangents obtained for the present EC cells are also in line with those of the reported values for LCs in the literature^23–26^, which lie in the range of $\tan ({\delta }_{perp})=0.05\ldots 0.075$ for the perpendicular state (OFF state) and in the parallel state (ON state) $\tan ({\delta }_{par})=0.03\ldots 0.05$. However, the tuneable range of the loss tangents of the present EC cells surpasses by a great margin the tunability of LC mixtures reported in the literature. The most distinguishing feature of the EC materials in this study is that their dielectric characteristics can be tailored (absolute and tunability values) without manipulating their chemical composition, as it is commonly done for almost all bulk tuneable materials, including LC. It is instructive to have a closer look the results of Fig. 6 and examine the origins of tunability of EC materials. The mechanism of dielectric tunability of EC materials is, effectively, a two-stage process. A simplified equivalent circuit of a basic EC cell is shown in Fig. 1(b). With reference to this figure, the total equivalent capacitance of the EC cell is the sum of the capacitances of the constituent layers, WO_3_, LiNbO_3_ and NiO, given by ${C}_{W{O}_{3}}$, ${C}_{LiNb{O}_{3}}$ and ${C}_{NiO}$, respectively, to yield

2 $${C}_{total}=\frac{{C}_{W{O}_{3}}{C}_{LiNb{O}_{3}}{C}_{NiO}}{{C}_{W{O}_{3}}{C}_{LiNb{O}_{3}}+{C}_{W{O}_{3}}{C}_{NiO}+{C}_{LiNb{O}_{3}}{C}_{NiO}}$$

In turn, these capacitances are proportional to their corresponding dielectric permittivities, thicknesses and surfaces areas by $C=\varepsilon \frac{A}{d}$. By EC cell actuation, ions and electrons migrate outside of the electrolyte layer and get injected into the chromic (WO_3_ and NiO) layers. The migration of the charges from the electrolyte structurally changes this layer and alters its dielectric characteristics. In particular, due to electrolyte charges depletion, the dielectric permittivity of this layer reduced, resulting in the reduction of the capacitance of this layer. These expelled ions and electrons are subsequently injected into the chromic, transition metal-oxide layers, located at the bottom and top electrodes. Their injection into these layers also changes their fundamental characteristics. Transition metal oxides, in general, behave as insulators, exhibiting values of resistivity high enough to be considered dielectrics. However, it has been shown^28^ that upon intercalation of Li ions into WO_3_, the resistivity of transition metals oxides exhibits a significant decrease, resulting in their transition from insulators to, relatively, poor conductors^27^ with a resistivity of up to 3 × 10^−3^(Ωcm), for values of x ~ 0.5 in Li_x_WO_3_. With reference to Figs 1(a) and 2(b) ion intercalation into the chromic layers results in an effective reduction of the height of the composite, non-conductive EC cell, i.e. h_EC_ = h_WO3_ + h_LiNbO3_ + h_NiO_~h_LiNbO3_ (500 nm for cell 1) and h_EC_ = h_WO3_ + h_LiNbO3_ + h_NiO_~h_LiNbO3_ (700 nm for cell 2), since the chromic layers in this case become, effectively, part of the top and bottom electrodes, respectively. The effect of reducing the height of the EC channel does not manifest itself in the increased losses only. This can be understood with reference to the relative dielectric permittivity of conductors

3 $${\varepsilon }_{rm}=1-j\frac{1}{\rho \omega {\varepsilon }_{0}}$$

where $\rho$, $\omega \,$ and ${\varepsilon }_{0}$ are the resistivity, frequency of operation and dielectric permittivity of vacuum, respectively. Since in the case of switched-on states, the resistivity of the chromic oxide layers decreases, this results in an increase of their relative dielectric permittivities, given by (3). This, in turn, increases the values of the corresponding, effective capacitances of the chromic layers, $\,{C}_{W{O}_{3}}$ and ${C}_{NiO}$. The modulation of the total capacitance, given by (2) can then be seen to be the product of two opposing effects. The increase of the dielectric permittivity of conductors is frequency dependent and is more pronounced at lower frequencies One possible way of reducing the effect of frequency dependence of the dielectric permittivity and increased losses lies with the reduction of the thicknesses of the oxide layers. This will have a minimal effect on the optical characteristics of the EC cells, due to its surface interface nature^12^. Furthermore, the reduction in the thickness of the oxide layers may result in the increase of dielectric tunability of the EC cell since the dielectric permittivity modulation of the electrolyte layer is not hampered by the opposing effects stemming from ion injection into the chromic layers. However, in this case, the dielectric permittivity of the EC cell is likely to be higher in the non-actuated state than in the actuate state.

On the other hand, the aforementioned effect of EC channel shortening can be enhanced, which can find use in switching applications. This can be done by reducing the thickness of the electrolyte and increasing the thicknesses of the chromic layers. In this case, in the off state, the EC cell may be considered as an open circuit with a height of h_EC_ = h_WO3_ + h_electrolyte_ + h_NiO_ and an associated open circuit capacitance dictated by the composite dielectric permittivity and channel height, whereas in the on state, the EC cell channel is shortened to h_EC_ = h_WO3_ + h_electrolyte_ + h_NiO_~h_electrolyte_ resulting in values of the equivalent capacitance high enough to be considered a short circuit. The exact values needed for this operation are, entirely dependent on the application and frequency of operation.

**Figure 7:**
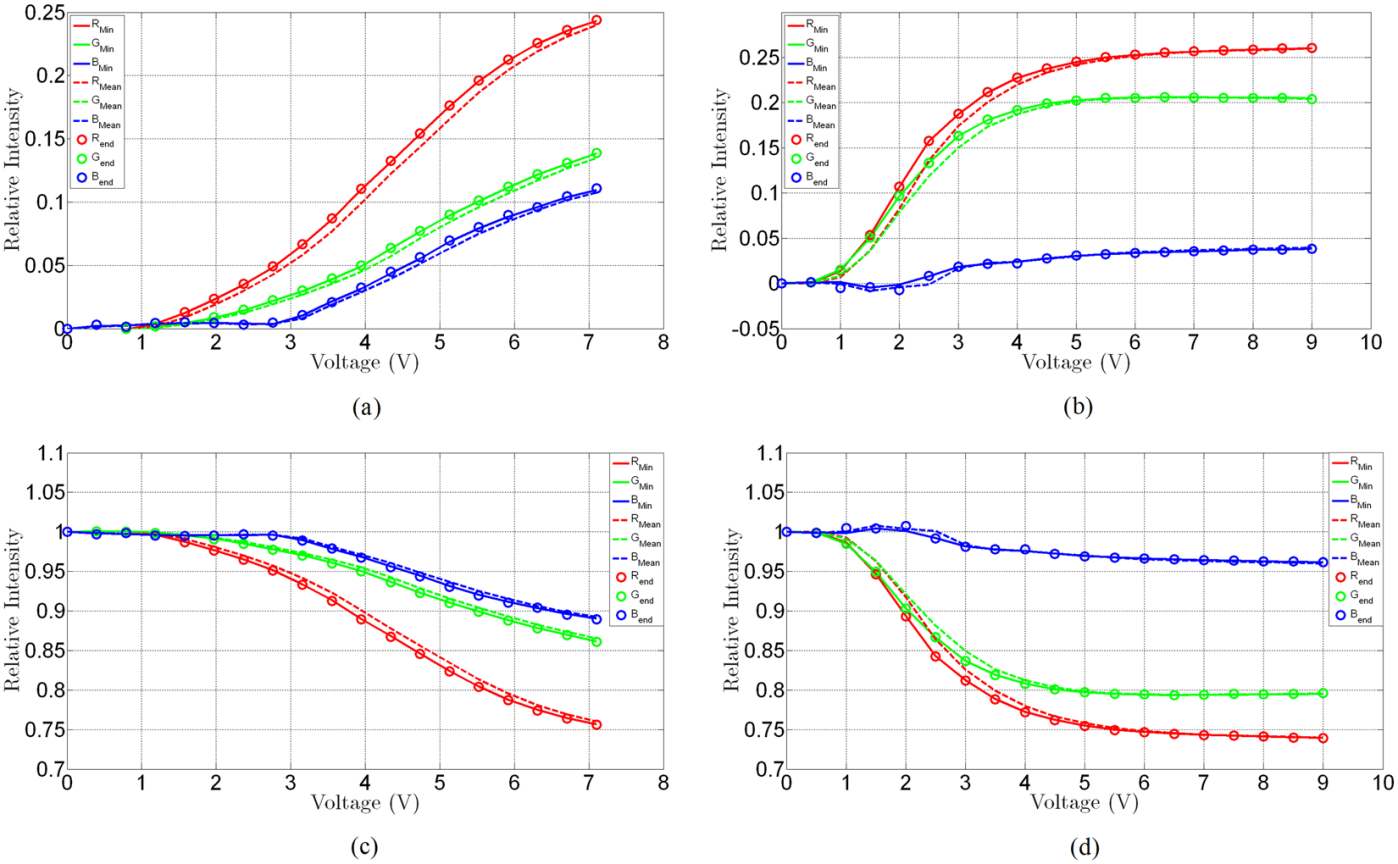
Decomposition of the optical response of EC cells into R-G-B colour components. Index “min” refers to the normalisation of the recorded minimum intensity of a particular colour component at a prescribed dc bias voltage applied for 6 minutes, index “mean” refers to the normalisation of the recorded average of a particular colour component at a prescribed dc bias voltage applied for 6 minutes and index “end” refers to the normalisation of the recorded end value of a particular colour component at a prescribed dc bias voltage applied for 6 minutes. (**a)** Cell 1, absorption spectrum, (**b)** cell 2, absorption spectrum, (**c)** Cell 1 reflection spectrum and (**d)**, cell 2 reflection spectrum.

The optical response of cells 1 and 2 is examined next, Fig. 7. For this purpose, the cells were actuated by gradually increasing the applied dc voltage from 0 to the maximum voltage– 7.1 V for cell 1 and 9 V for cell 2. Here, the dc bias voltage is increased from 0 V to the maximum voltage in 17 steps (limitation of the equipment). For cell 1, this step is 0.41 V and for cell 2 it is 0.52 V. At each dc bias voltage step, the cells are kept at the prescribed dc bias voltage for 6 minutes and a micrograph of the cell is taken every 15 seconds, while the cells were illuminated using a white light source. The videos obtained from the composition of the images obtained in this way are available as the Supplementary files (for cell 1 – “Video-cell 1” and for cell 2 – “Video- cell 2”). The images of the EC cells obtained in this way are then analysed and decomposed into the Red-Green-Blue (RGB) components. Each component is then normalised to its initial value at 0 V, to obtain relative changes in optical intensity. In this regard, for each colour component three cases are distinguished; (i), at each dc bias voltage step the light intensity of a particular colour component is averaged over the time it was exposed to the particular bias voltage (6 minutes) and then normalised to its value at 0 V; (ii), at each dc bias voltage step, the maximum (end value) light intensity of a particular colour component recorded for the duration (6 minutes) of its exposure to a particular dc bias voltage level is normalised to its value at 0 V and (iii), at each dc bias voltage step, the minimum light intensity of a particular colour component recorded for the duration (6 minutes) of its exposure to a particular dc bias voltage level is normalised to its value at 0 V. This is done so that the speed of colour change is adequately recorded.

In general, the decomposed optical responses vs. applied dc bias voltages of these two cells feature different characteristics. Three principal observations can be made regarding this figure: First, cell 1 begins to switch on at a dc bias voltage of about 1.5 V, while the threshold voltage for cell 2 occurs at a dc bias voltage of above 1 V. Second, a naked-eye inspection of the cells of Fig. 5 suggests that the cells turn blueish by dc bias voltage actuation, however, the decomposition of the optical response of the EC cells suggests that, in comparison with the R and G component, the B component remains relatively unaffected by the actuation. Third, cell 1 in contrast to cell 2, appears not to have fully switched on, even after a long exposure to the actuation voltage, suggesting that the reduction in the height of the electrolyte layer has resulted in a reduced number of available ions and electrons needed to induce this change.

The results presented in this work indicate an intricate relationship between optical properties of EC materials and their dielectric properties in the mm-wave domain. Both of the observed phenomena can be attributed to the same processes in the electronic/ionic structure of the underlying electrochromic material. Coupled with the fact that these responses can be tailored without changing material composition it is likely to enable a wide variety of devices capable of exploiting this effect – both in the mm-wave and optical domains. Some examples pertain to tunable mm-wave components (filters and antennas), but also tunable optical devices (optical filters). Furthermore, the findings in this work are likely to enable a new class of devices capable of exploiting the linkage between the mm-wave and optical fields. Of course, this effect needs to be further investigated in the context of other transition metal oxides in order to gain a thorough understanding of the matter.

## Discussion

In this paper, we have demonstrated that the extent of tunability of dielectric and optical properties of EC materials with electrochromical complementary Conductor/WO_3_/LiNbO_3_/NiO/Conductor structure can be varied without changing their material composition. For this purpose, two inorganic EC cells, differing only in the height of the electrolyte layer (500 nm for cell 1 and 700 nm for cell 2), are used. The high frequency dielectric characteristics of the proposed cells are extracted in a systematic way in the frequency range from 1 GHz to 20 GHz for the non-actuated and actuated states respectively. The actuation is achieved by the application of a dc bias voltage on the EC cells through a purposefully designed CPW-to-microstrip transition. The extracted dielectric characteristics of the two cells show that the variation in the height of the electrolyte layer did not only result in their relative, percentage change (between non-actuated and actuated states), but also in the changes of their absolute values. Further, the decomposition of the optical responses (absorbance and reflectance) of the two EC cells reveals different colour composition of the cells and different actuation dc bias voltages.

A short comparison of the present finding on the EC materials having a complementary structure of conductor/WO_3_/LiNbO_3_/NiO/Conductor with a more mature, state-of-the-art Liquid Crystal (LC) bulk tunable technology is also presented. The results indicate that while the percentage tunability of the dielectric permittivity of the present EC materials is similar to those of LCs, their absolute values of the dielectric permittivity are somewhat higher, but can be tailored-made. This is an important advantage of EC materials, since the dielectric characteristics of LC media is not possible to be height-tailored.

The findings reported in this work give rise to the possibility of creation of a new class of devices capable of exploiting pairing dielectric and optical effects. There are many examples, ranging from thin displays with a tailor-made optical response and beam-forming function to tuneable optical and microwave devices, such as phase shifters, switches, attenuators and antennas.

## Electronic supplementary material

Video-cell 1

Video-cell 2
